# Imperfect hydroxyapatite bioceramics derived from golden pomfret have enhanced osteogenic properties

**DOI:** 10.1038/s41598-025-06015-8

**Published:** 2025-07-01

**Authors:** Changze Zhang, Guangchun Zhao, Xiaorui Wang, Mengting Li, Zhengmao Li, Yixun E, Xiaxin Cao, Maohua Chen, Chaozong Liu

**Affiliations:** 1 School of Chemistry and Chemical Engineering, Hainan University, Haikou, 570228 China; 2 Laboratory of Digital Medical Engineering, Key Laboratory of Biomedical Engineering of Hainan Province, School of Biomedical Engineering, Hainan University, Sanya, 572025 China; 3 School and Hospital of Stomatology, Guangzhou Medical University, Guangzhou, 510182 China; 4 Institute of Orthopaedic & Musculoskeletal Science, University College London, Royal National Orthopaedic Hospital, London, HA74LP UK

**Keywords:** Calcium-deficient hydroxyapatite, Carbonate substitution hydroxyapatite, Cation exchange, Enhanced osteogenic property, Golden pomfret bone, Biomedical materials, Biomedical engineering

## Abstract

Imperfect hydroxyapatite (IHA) bioceramics, which contain defects such as calcium deficiency, carbonate substitution, and metal cation substitution, exhibit improved osteogenic properties. In this study, we used a two-step calcination-hydrothermal process to manufacture two types of golden pomfret bone-derived imperfect hydroxyapatite bioceramics (G-IHA): carbonated calcium-deficient hydroxyapatite (CD-IHA) and carbonated hydroxyapatite (C-IHA). Their composition, surface morphology, zeta potential, degradation capacity, mineralization and osteogenic properties were systematically investigated. The results revealed that G-IHA with a higher defect content, including A-type carbonate substitution and Ca vacancies, had negatively charged surface. As a result, G-IHA surfaces are more favourable to ion exchange and interaction with cations (e.g., Na^+^, Ca^2+^) in the microenvironment, which results in improved degradation and mineralization. Specifically, after 28 days of degradation, G-IHA showed significantly higher weight losses (CD-IHA and C-IHA were 17% and 13%, respectively) than commercial hydroxyapatite (CHA; 7%). In addition, G-IHA have a higher better bone-like apatite formation ability, and a higher degree of osteogenic differentiation than CHA. Notably, carbonated calcium-deficient imperfect hydroxyapatite (CD-IHA) exhibited the highest bioactivity and osteogenic capacity as evidenced by its increased alkaline phosphatase activity and improved bone matrix mineralization capacity. In conclusion, this study revealed that imperfect hydroxyapatite bioceramics derived from golden pomfret bone have the potential to enhance osteogenic properties and be employed in clinical settings as bone substitute materials.

## Introduction

The Global Burden of Diseases, Injuries, and Risk Factors Study indicated that the number of prevalent cases of acute or long-term symptoms of a fracture had reached 455 million by 2019, representing a 70.1% increase compared with 1990^1^. Although bone tissue can regenerate to some extent, when bone deficiencies are larger than the critical size and are induced by external sources, including fractures, bioactive bone transplants are required to promote bone regeneration^2^. Hydroxyapatite (Ca_10_(PO_4_)_6_(OH)_2_), a calcium phosphate (CaP) compound, has been extensively employed as a bone transplant material because of its superior osteoinductive activity^3^. The majority of hydroxyapatite found in natural bone is calcium-deficient (Ca_10 − x_(HPO_4_)_x_(PO_4_)_6−x_(OH)_2−x_, 0 < x < 1, CDHA)^4^;^5^.

Compared with HA, CDHA, whose amorphous surface layer provides a metastable non-apatite environment, promotes ion exchange on its surface, resulting in superior bioactivity^6–8^. Zhang et al. investigated the effects of HA and CDHA on mouse bone mesenchymal stem cells (BMSCs). The results indicated that CDHA exhibited more favourable effects than HA in terms of cell proliferation, alkaline phosphatase activity, and expression of osteogenesis-related genes^9^. After evaluating the osteogenic potential of HA and CDHA in vivo, Bulina et al. observed that CDHA exhibited almost complete remodelling of injured rat bone tissue after just 5 months, whereas HA only showed partial remodelling^10^.

Compared to pure HA, carbonated HA, which facilitates the release of BMP6 and Wnt10b, can promote osteoblastic differentiation^11–13^endowing carbonated HA with superior osteogenic activity. Fish bones are an excellent source of carbonated hydroxyapatite for bone graft materials^14^;^15^. Shi et al. demonstrated that carbonated hydroxyapatite originating from rainbow trout or salmon bones can enhance mouse pre-osteoblast MC3T3-E1 proliferation and differentiation^16^. Deng et al. found that carbonated biphasic CaP derived from butterfish bones possesses superior biocompatibility and osteogenic potential in vivo and in vitro^17^.

The golden pomfret fish (Trachinotus ovatus) is cultivated in China, Singapore, and Malaysia^18^. The annual production of golden pomfret in southern China is 120 thousand tons^19^. However, research on golden pomfret has been mainly focused on its cultivation or processing for food^20–22^. Bone-derived biomaterials have received little attention.

In this study, golden pomfret bone was used to generate two types of imperfect hydroxyapatites, carbonated calcium-deficient imperfect hydroxyapatite (CD-IHA) and carbonated imperfect hydroxyapatite (C-IHA), through a two-stage hydrothermal calcination process. Initially, the phase composition, crystal structure, surface charge properties, mineralization capacity, and degradation rates of the CD-IHA, C-IHA and CHA bioceramic samples were investigated to characterize the properties of the bioceramics derived from golden pomfret bone. Subsequently, the in vitro biological performance of the bioceramic samples was evaluated to confirm their osteogenic performance and biomaterial applications, including phosphate buffered saline (PBS) buffer immersion, bone-like apatite formation, cell viability, and osteoblastic differentiation of BMSCs.

## Materials and methods

### Materials

The raw material used in this study was golden pomfret purchased from Hainan Xiangtai Fishery Company Limited, Chengmai, Hainan Province, China. As a control, CHA was obtained from Wuhan Lullaby Pharmaceutical Chemical Company, Ltd. (Wuhan, Hubei Province, China). Furthermore, the simulated body fluid (SBF) solution was prepared as described by Oyane et al.^23^but at double the concentration.

### Preparation of imperfect hydroxyapatite bioceramics derived from golden pomfret

**Fig. 1:**
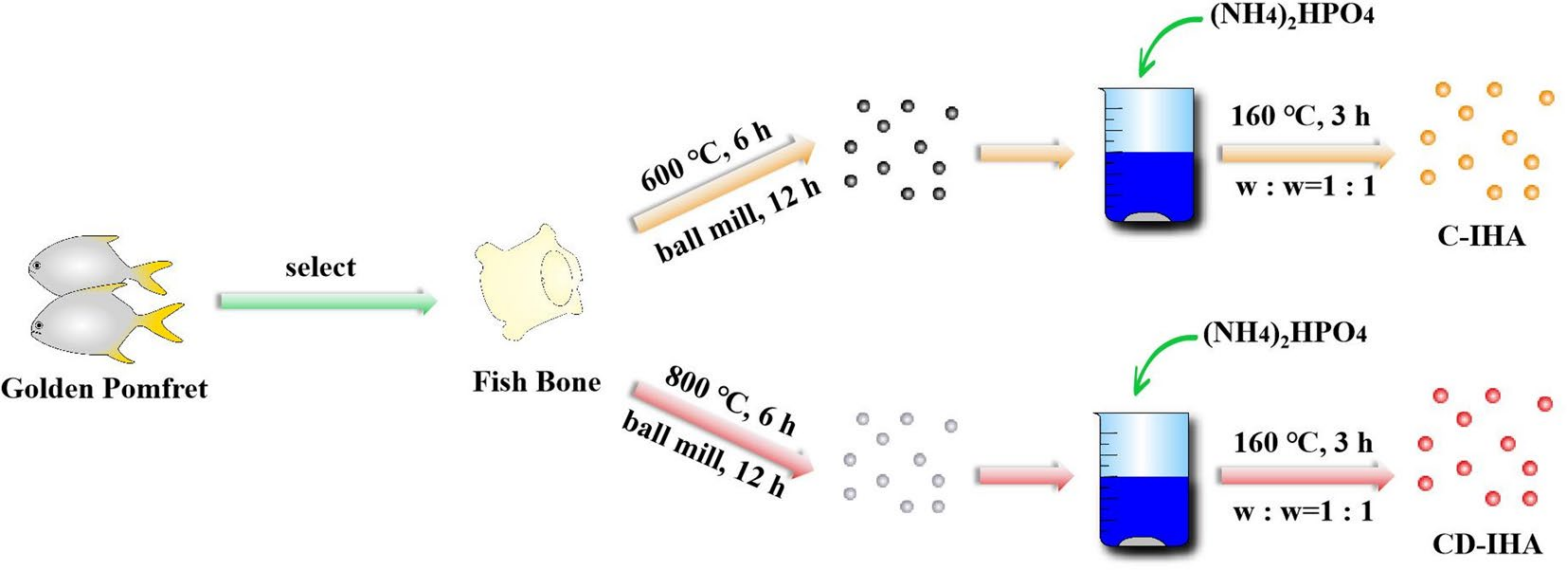
Process of preparing bioceramics derived from golden pomfret.

The golden pomfret bones were calcined at 800 °C for 6 h in an air atmosphere and subjected to ball milling for 12 h. Subsequently, the ball-milled bones were mixed with ammonium phosphate dibasic ((NH_4_)_2_HPO_4_) at a mass ratio of 1:1 and added in deionized water, maintaining at 160 °C for 3 h to obtain CD-IHA. When the temperature of calcination was elevated to 600 °C, C-IHA was obtained (Fig. 1).

### Characterization

The phases of the samples were analyzed by X-ray diffraction (XRD) analyzer (Smart Lab, Rigaku, Japan) using Cu Kα1 radiation at 40 kV in the 2θ range of 10-80°, and the crystal structure data was calculated by Rietveld method for lattice parameters calculation^24^. The crystallinity was calculated according to Alain Person’s research^25^. For composition characterization, a Fourier-transform infrared (FTIR) spectrometer (T27, Bruker, Germany) was used in the range of 4000–400 cm^–1^. Derivative thermogravimetry (DTG) graphs of the samples were recorded using thermogravimetric analysis (TGA) data. The analyses were carried out on a thermogravimetric analyzer (STA449F5, Netzsch, Germany) at 25–1100 °C with a heating rate of 10 °C/min. An energy dispersive spectrometer (EDS) attached to a scanning electron microscope (SEM) at 3 kV was used for morphological and elemental analyzes. The surface charge of the samples was measured using a zeta potential analyzer (Zetasizer Nano ZS90, Malvern Instruments, Britain) at a suspension concentration of 12.5 mg/mL.

### In vitro studies

#### Degradation

Samples were immersed in PBS to be oscillated at 37 °C for 7, 14, 21 and 28 days at a concentration of 1 mg/mL each. Each suspension was centrifuged for 1 min at 6577 x g. Finally, the pH of the supernatant was analyzed, and the mass of precipitate that had been dried at 60 °C was quantified. Each experiment was conducted in triplicate.

About degradation rate, the weight loss (%) was calculated by the Eq. (1):

1 $${\text{w}}(\% )=\frac{{{{\text{m}}_{\text{0}}} - {\text{m}}}}{{{{\text{m}}_{\text{0}}}}}{{ \times 100\% }}$$

Where, w is the degradation rate of the sample, m_0_ is the initial mass of the sample, and m is the mass of the corresponding precipitate.

#### Mineralization

Each sample was immersed in SBF and oscillated at 37 °C at a concentration of 1 mg/mL. The SBF was updated every 7 days to ensure an adequate ionic concentration for mineral nucleation and growth. After 14-days of immersion, the precipitate was separated and dried at 60 °C. The composition and morphology of the precipitate was analyzed by XRD, FTIR and SEM by the method in section “Characterization” of this article.

#### Cytocompatibility

The L929 fibroblasts were cultured with a medium (RPMI-1640 supplemented with 10% fetal bovine serum and 1% penicillin-streptomycin mixture) containing various concentrations of samples (0 µg/mL (control), 400 µg/mL, 600 µg/mL, 800 µg/mL and 1000 µg/mL) for 24 h. The BMSCs were cultured under the same conditions, except that the medium was mainly DMEM-F12 instead. Subsequently, cell viability was measured by replacing the complete medium with 100 µL/mL cell counting kit-8 reagent (CCK-8; Dojindo, Japan) and culturing for another 1 h. The optical density (OD) of each well was analyzed using a microplate reader (Synergy LX, Biotek, USA) at 450 nm. The survival of the cells co-cultured with the samples was calculated according to Eq. (2) ^26^:

2 $${\text{Sur(\%)}}=\frac{{{{\text{A}}_{\text{s}}} - {{\text{A}}_{\text{0}}}}}{{{{\text{A}}_{\text{c}}} - {{\text{A}}_{\text{0}}}}}{{ \times 100\% }}$$

where Sur represents the survival of cells cultured with samples, A_s_ represents the OD of wells with the sample, A_c_ represents the OD of the control, and A_0_ represents the OD of the blank. A blank group was established using the corresponding complete medium without cells.

At the same time, cell proliferation was further evaluated by live/dead staining. Briefly, the cultured wells were stained using calcein-AM/PI kit (Sigma Aldrich, USA) for 30 min and imaged under a microscope.

#### Osteogenic ability

The osteogenic ability of the samples was assayed using alkaline phosphatase (ALP) and Alizarin red S (ARS) staining. For ALP or ARS staining, BMSCs were co-cultured with 1 mg/mL samples by the method described in section “Cytocompatibility” for 7 or 14 days, respectively. Subsequently, each well was fixed with 4% paraformaldehyde (Service Bio, China) and cultured with a BCIP/NBT ALP colour development kit (Beyotime, China) or ARS solution (OriCell, China) at about 25 °C for 30–10 min, respectively. Thereafter, the cells were imaged under a microscope. The ALP- and ARS-stained areas were calculated to quantify their corresponding activities^27–29^ using Python 3.11.

### Statistical analysis

In this study, Microsoft Excel 2019 was employed to conduct pairwise F-tests and t-tests on the data. Specifically, when *p* > 0.05, a heteroscedasticity t-test was performed on the paired data. Conversely, when *p* < 0.05, a homoscedasticity t-test was conducted on the paired data using the F-test. Statistically significant differences between the two groups are indicated by * (*p* < 0.05), ** (*p* < 0.01), or *** (*p* < 0.001) in the t-test.

## Results and discussion

### Composition

**Fig. 2:**
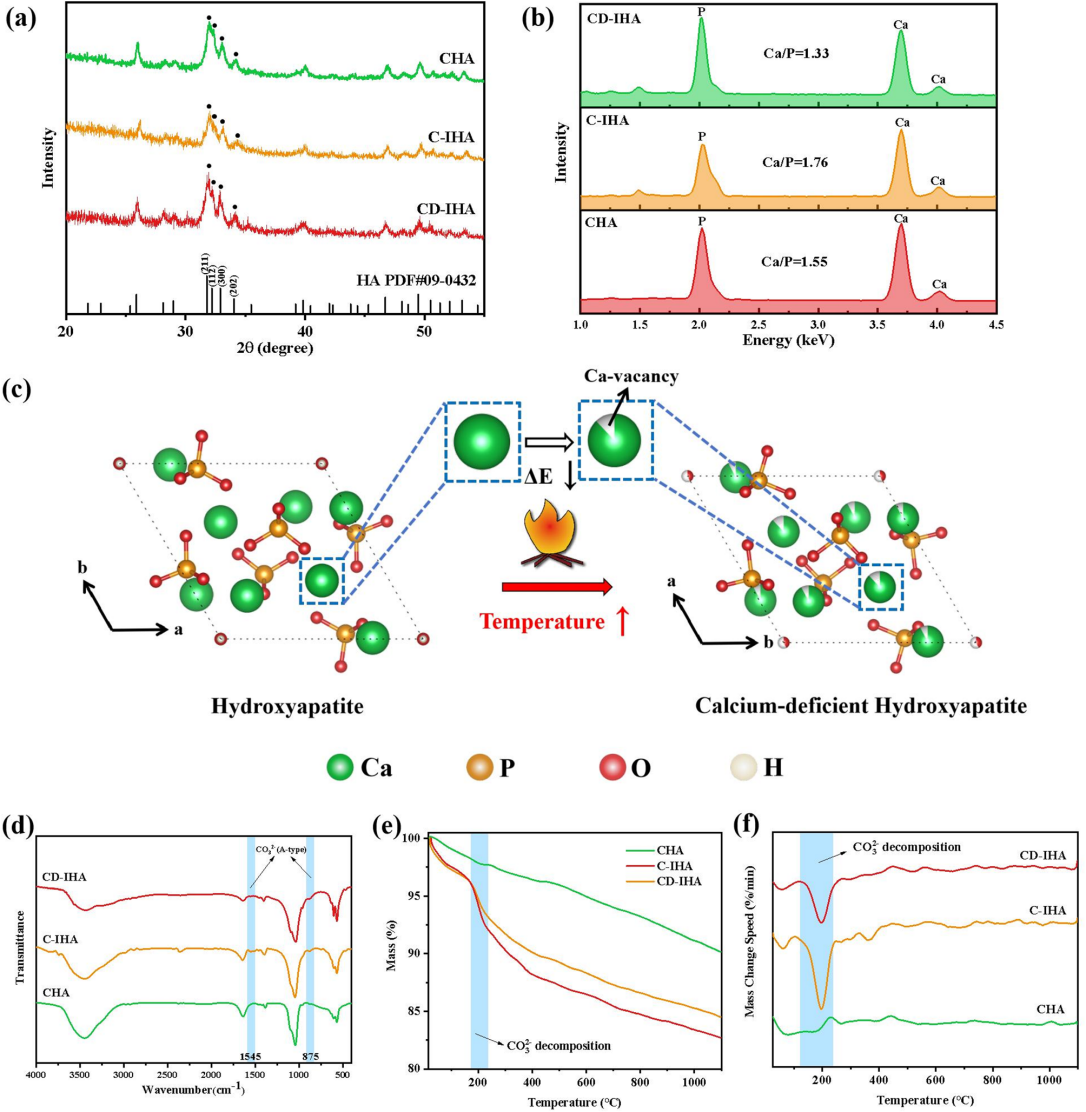
(a) XRD pattern of CHA, C-IHA and CD-IHA; (b) EDS spectra and the calculated Ca/P ratio of CHA, C-IHA and CD-IHA ; (c) Mechanism of calcium vacancy formation in the HA structure above 800 °C (analyzed by VESTA software^69^); (d) FTIR spectra of CHA, C-IHA, and CD-IHA; (e) TG graphs of CHA, C-IHA, and CD-IHA; and (f) DTG graphs of CHA, C-IHA, and CD-IHA calculated by TG data.

Golden pomfret bone-derived imperfect hydroxyapatite bioceramics (G-IHA) were prepared using a two-step calcination-hydrothermal method. About the crystal phase, XRD peaks at 32.0°, 32.4°, and 33.1°, which were individually identified as the (211), (112) and (300) reflections of HA (ICDD PDF No. 09-0432)^30^were observed for both G-IHA and CHA (Fig. 2.a). G-IHA prepared by calcining golden pomfret exhibits a higher crystallinity (~ 85%; Table 1).

**Table 1:** Crystal structure data of the standard HA and samples.

Samples	a (Å)	c (Å)	Crystallinity (%)
Standard HA	9.418	6.884	-
CHA	9.394	6.885	58.64
C-IHA	9.416	6.893	85.12
CD-IHA	9.421	6.878	88.91

Furthermore, the Ca/P ratio of CD-IHA was determined to be 1.33, which is lower than that of standard hydroxyapatite (HA), carbonate-hydroxyapatite (CHA), and carbonated hydroxyapatite (C-IHA), with values of 1.67, 1.55, and 1.76, respectively (Fig. 2.b). As for previous research, when the Ca/P ratio approaches 1.4, calcium vacancies are formed within the hydroxyapatite lattice^31^. This finding provides additional evidence for the presence of calcium vacancies in CD-IHA. Conversely, C-IHA does not display any calcium vacancies in its structure, a difference that can be attributed to the distinct calcination temperatures employed during their synthesis.

Due to the lower sintering temperature of C-IHA, there are no obvious calcium vacancies in its structure. As shown in Fig. 2.c, metallic vacancy formation energy (ΔE) in materials is negatively correlated with calcination temperature at constant thermodynamic conditions, including the boiling point, molar heat of evaporation, specific heat at constant volume and temperature profiles^32^. Therefore, the higher the calcination temperature of the same material, the easier the formation of metallic vacancies.

The FTIR spectra of G-IHA exhibited two absorption bands at 875 cm^–1^ and 1545 cm^–1^ (Fig. 2.d). These bands are attributed to A-type carbonate substitution in the apatite structure, where the hydroxyl group (OH^−^) was replaced by carbonate ions^33^. The obtained results were consistent with previous findings^34–36^. TG and DTG analysis were conducted to further investigate the carbonate substitution in G-IHA. In comparison to CHA, a distinct weight loss was observed in G-IHA at approximately 200 °C in G-IHA (Fig. 2.e and 2.f). This is attributed to the generation of CO_2_ in carbonated HA at roughly 200 °C^37^. According to previous research, the reactions can be described by the Eqs. (3) and (4)^38,39^.

3 $${\text{CO}}_{{\text{3}}}^{{{\text{2}} - }}{\text{+}}{{\text{H}}_{\text{2}}}{\text{O}} \to {\text{HCO}}_{{\text{3}}}^{ - }{\text{+O}}{{\text{H}}^ - }{\text{~}}$$

4 $${\text{HCO}}_{{\text{3}}}^{ - } \to {\text{C}}{{\text{O}}_{\text{2}}} \uparrow {\text{+O}}{{\text{H}}^ - }{\text{~}}$$

Therefore, G-IHA was successfully prepared through calcination at 600 °C or 800 °C with subsequent hydrothermal treatment. Both were carbonated, with CD-IHA, possessing Ca vacancies.

### Surface charge

**Fig. 3:**
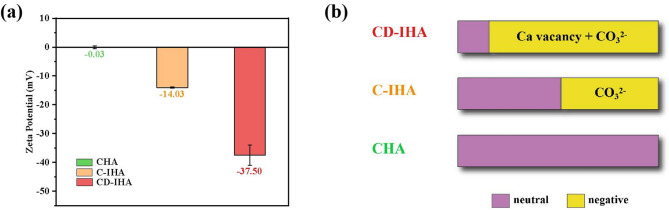
(a) Zeta potentials of CHA, C-IHA and CD-IHA; and (b) sources of charges in CHA, C-IHA and CD-IHA.

A negatively charged surface promotes the deposition of apatite and enhances the adhesion of pre-osteoblasts, thereby facilitating bone mineralization and osteoblastic differentiation^40,41^. As a result, the surface charges of the samples were determined by their zeta potentials^42^. The results in Fig. 3.a showed that zeta potentials of CHA, C-IHA and CD-IHA are − 0.03 mV, − 14.03 mV and − 37.50 mV, respectively. This suggested that the surfaces of G-IHA were negatively charged, while the surfaces of CD-IHA had more of negative charges than the surfaces of C-IHA. The phenomenon may be attributed to the carbonate substitution of imperfect hydroxyapatite, which elevates the concentration of negatively charged groups on the C-IHA surface^43^. In the meantime, the negative charges will be increased by an abundance of Ca vacancies in the CD-IHA^44,45^.

Consequently, G-IHA exhibited a negative charge due to carbonate substitution as well as calcium vacancies inside its lattice structure. (Fig. 3.b).

### In vitro degradation

**Fig. 4:**
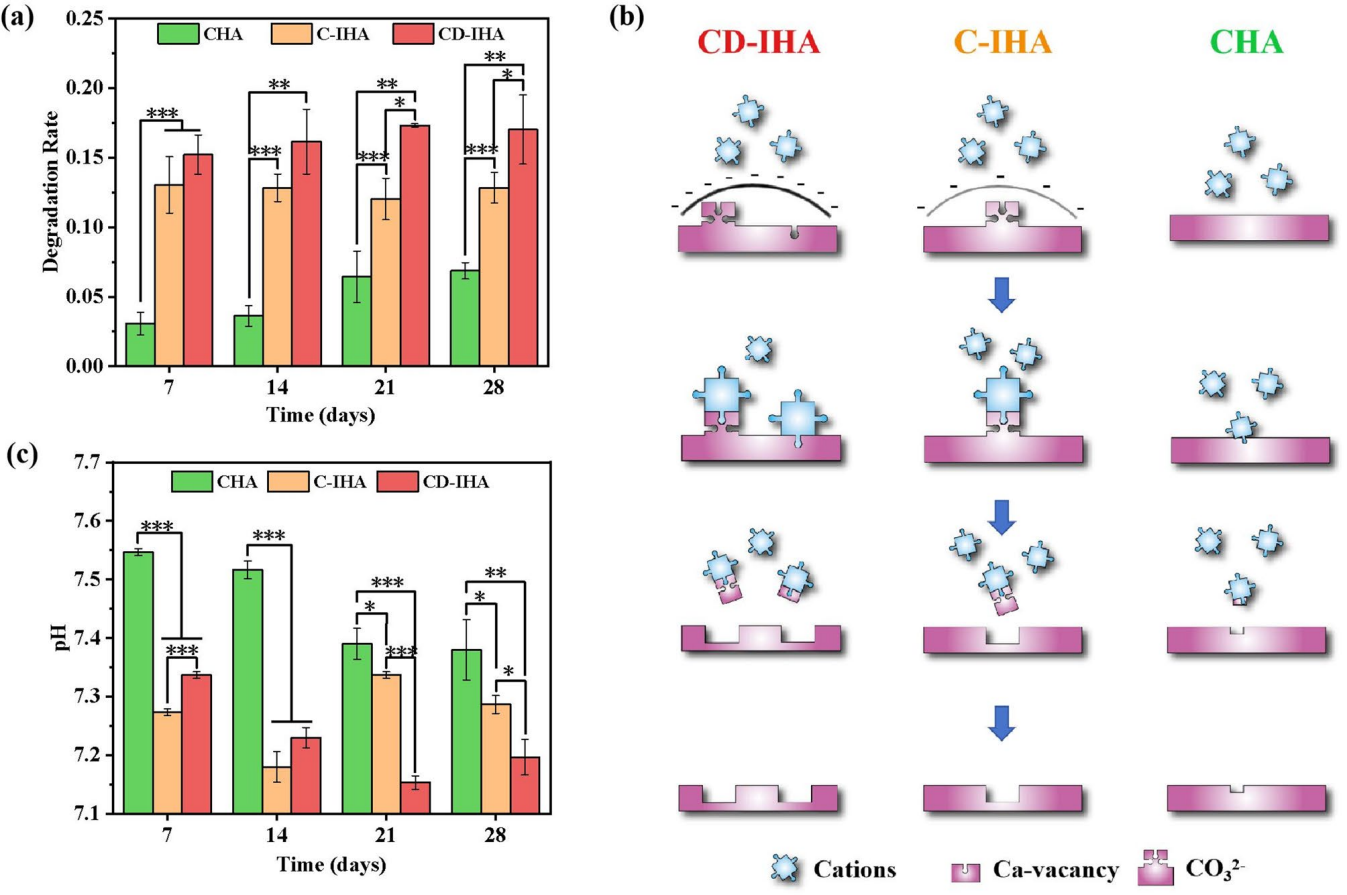
(a) Degradation rate of CHA, C-IHA, and CD-IHA in vitro; (b) degradation mechanism of CHA, C-IHA, and CD-IHA in vitro; and (c) pH variation during the degradation of CHA, C-IHA, and CD-IHA in vitro.

Degradation performance is a key indicator in the evaluation of bone grafts^2^. Therefore, the degradation rates of CHA and golden pomfret bone-derived imperfect hydroxyapatite bioceramics (G-IHA) were evaluated in PBS for 28 days. After 28 days of immersion, the degradation rates of CD-IHA, C-IHA and CHA reached 17%, 13% and 7%, respectively (Fig. 4.a). Although G-IHA has higher crystallinity than CHA as reported in Table 1, its degradation rate is accelerated due to crystal defects and carbonate substitution.

The degradation of G-IHA is significantly influenced by the ion exchange process of cations across the degradation zone^46^. The cations in PBS, primarily Na^+^ and K^+^, can be readily absorbed by the negatively charged G-IHA^47^. Subsequently, the anions on the G-IHAs interact with these cations, resulting in the formation of soluble salts (sodium or potassium salt) that dissolve in PBS solution and promote a loss of mass in the G-IHAs (Fig. 4.b). Furthermore, the crystal defects in G-IHA, resulting in irregular grain boundaries, also accelerate their degradation^48,49^. Simultaneously, the amorphous surface layer of CD-IHA, which facilitates ion exchanges via the metastable non-apatite surface, may further enhance the degradation rate^6–8^. Therefore, CD-IHA with higher negative charges exhibited improved degradation properties as compared to the C-IHA and CHA (*p < 0.05). (Fig. 4.a and b).

In addition, during the degradation process of all samples, the pH of the corresponding samples was maintained within the range of 7.15–7.55 (Fig. 4.c), which is similar to the pH observed in the environment of the human bone^50^.

### In vitro mineralization

Mineralization test in vitro is capable of identifying the reliably osteoinductive materials^51^. Therefore, it is important to evaluate the in vitro mineralization properties of bone grafts.

**Fig. 5:**
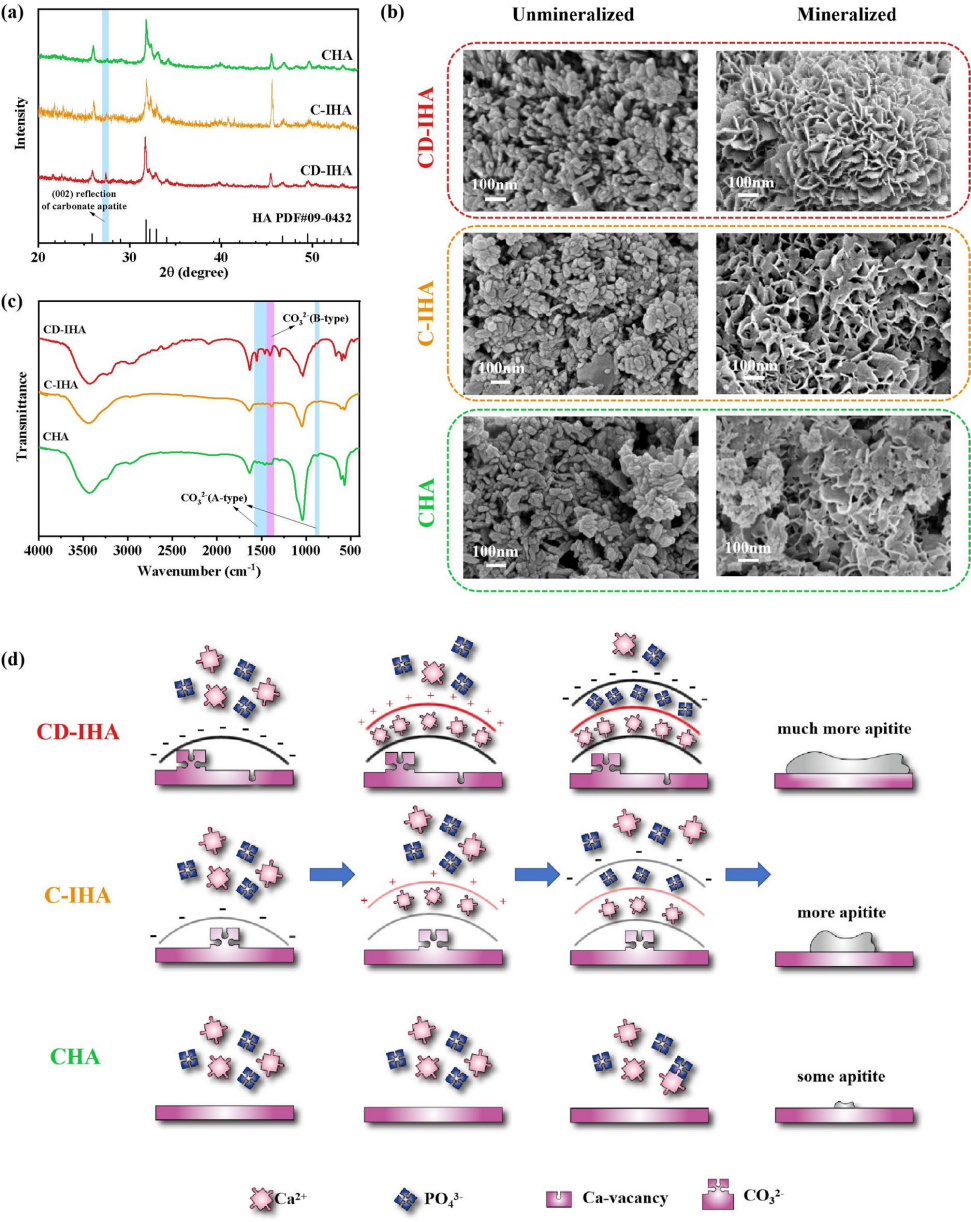
(a) XRD pattern of CHA, C-IHA and CD-IHA after immersion in SBF; (b) SEM images of CHA, C-IHA and CD-IHA before and after immersion in SBF; (c) FTIR spectra of CHA, C-IHA and CD-IHA after immersion in SBF; and (d) mineralization mechanism of CHA, C-IHA and CD-IHA during immersion in SBF.

XRD results indicated that both CHA and golden pomfret bone-derived imperfect hydroxyapatite bioceramics (G-IHA) had an increase in major peak intensity and a decrease in half-weight width after 14 days of immersion in SBF (Fig. 5.a). This is a result of the deposition of new apatite on the surface of both samples^52,53^. Furthermore, new peaks appeared in both immersed CHA and G-IHA at 27.4° (Fig. 5.a), which could be attributed to the (002) reflection of the deposited carbonate hydroxyapatite^54^. According to previous studies, the peak at 46° corresponds to the (221) reflections of aragonite commonly formed by Ca^2+^ and CO_3_^2−^ in SBF^55^.

Moreover, SEM results (Fig. 5.b) showed that the morphology of immersed G-IHA transferred into flower-like, indicating that G-IHA had apatite deposited on its surface. In addition, a new band at 1409 cm^− 1^ appeared in the FTIR spectra of immersed samples (Fig. 5.c), suggesting that CO_3_^2−^ substituted PO_4_^3−^ and B-type CO_3_^2−^ developed in their deposited apatite structure^56,57^. The B-type CO_3_^2−^ is predominant form of CO_3_^2−^ in human bone^58^suggesting that the deposition on the samples appeared like bone-like apatite. Notably, CD-IHA exhibited a stronger band than C-IHA and CHA (Fig. 5.c), suggesting enhanced deposition of bone-like apatite on CD-IHA.

The process of mineralization is associated with ion interaction^47^. In a manner similar to degradation, the negatively charged surface with apatite structure, having high binding affinity, preferentially attracts cations (such as Ca^2+^) from SBF solution. As a consequence, the calcium-rich surface attracts PO_4_^3−^ from the SBF, resulting in a cation-deficient surface that has the potential to continue attracting Ca^2+^^40^. Being negatively charged, G-IHA perform superior mineralization properties than CHA. Furthermore, since CD-IHA takes advantage of ion interaction more than C-IHA^6–8^more Ca^2+^ and PO_4_^3−^ ions are attracted to develop a new layer of apatite^40^resulting in enhanced mineralization properties (Fig. 5.d). Overall, CD-IHA demonstrated superior mineralization properties compared to other bioceramics.

### Cytocompatibility

**Fig. 6:**
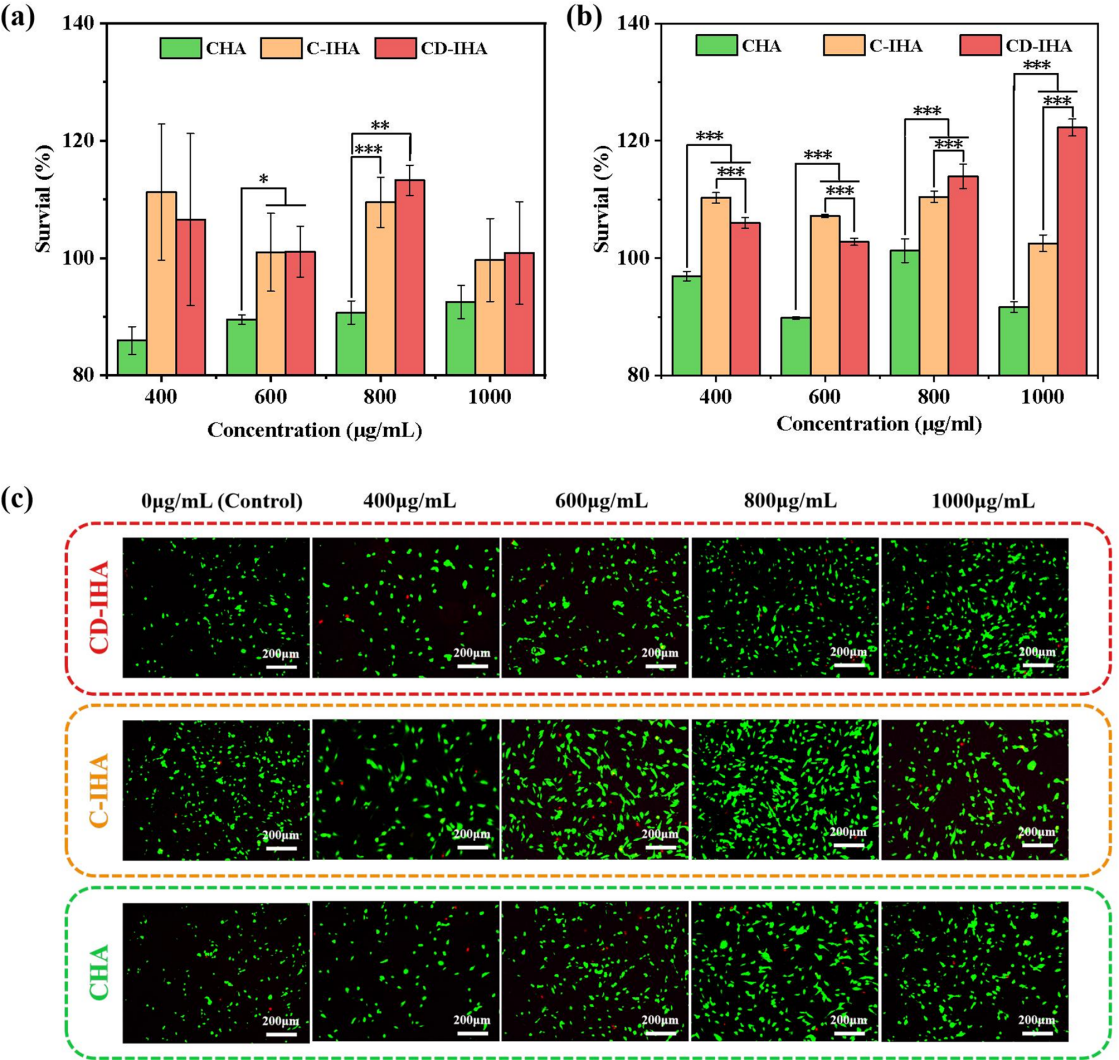
(a) Cell viability of L929 co-cultured with CHA, C-IHA and CD-IHA at various concentrations; (b) BMSCs co-cultured with CHA, C-IHA and CD-IHA at various concentrations; (c) Calcein-AM/PI staining result of BMSCs co-cultured with CHA, C-IHA and CD-IHA at various concentrations.

Cytocompatibility is an important consideration in bone-tissue engineering^59^. Therefore, we evaluated the cytocompatibility of CHA and golden pomfret bone-derived imperfect hydroxyapatite bioceramics (G-IHA). According to the ISO 10993-5 standard, if the viability of the cells falls to < 70%, it is considered toxicity^60^. As shown in Figs. 6.a and 6.b, the cell viability was > 80% following the co-cultivation of L929 cells or BMSCs with CHA or G-IHA at varying concentrations. The live/dead staining results of the BMSCs (Fig. 6.c) also agreed with those of the quantitative evaluation, indicating that both CHA and G-IHA are biocompatible. Consequently, both the CHA and G-IHA exhibited excellent cytocompatibility.

### Osteogenic ability in vitro

Bone grafts are more commonly used in situations similar to a suspension, that is, where the particles are surrounded by body fluid at the defect site in vivo, rather than extracts^61^. Consequently, BMSCs were co-cultured with the suspension in this study to evaluate the osteogenic ability of the samples.

**Fig. 7:**
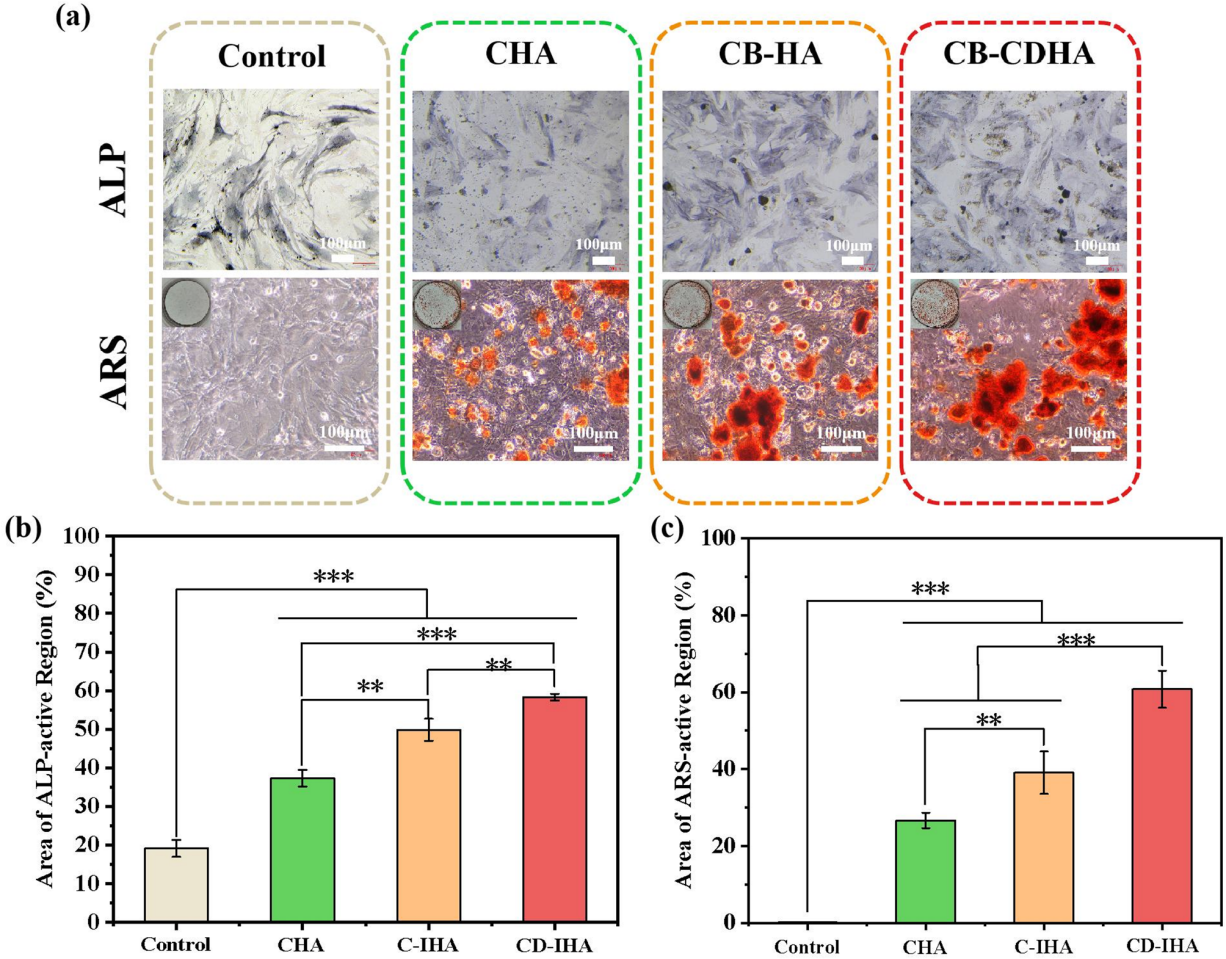
(a) ALP and ARS staining image of CHA, C-IHA, CD-IHA, and control group; (b) area of ALP-active region of CHA, C-IHA, CD-IHA, and control group; (c) area of ARS-active region of CHA, C-IHA, CD-IHA, and control group.

ALP is often used as an early stage marker of osteogenesis^62^. This is shown in Fig. 7.a and 7.b, where it could be seen that the ALP activity in CD-IHA and C-IHA reached 58% and 49%, respectively, which was significantly higher than that in CHA (37%, **p < 0.01). Additionally, the CD-IHA group exhibited significantly higher ALP activity than the C-IHA group (** *p* < 0.01; Figs. 7.a and 7.b). ARS is a late-stage marker of extracellular matrix mineralization^62^. After 14 days of co-cultivation, ARS activity in CD-IHA and C-IHA reached 61% and 39%, respectively, which was significantly higher than that in CHA (27%, ***p < 0.001 for CD-IHA and **p < 0.01 for C-IHA) (Fig. 7.a and c). Furthermore, the CD-IHA group exhibited significantly higher ARS activity than the C-IHA group (*** *P* < 0.001; Fig. 7.a and c). These results indicate that the suspension of imperfect hydroxyapatite provides a more favorable environment for osteoblastic differentiation and CaP mineralization in BMSCs^28,63,64^. According to related researches, negative surface of apatite has a higher binding affinity to calcium ions and is capable of enriching them^47^. As a result, G-IHA can facilitate the proliferation and differentiation of osteoblasts via improved ion exchange and interaction, thereby stimulating the subsequent bone formation process^41,47,65^.

Ion doping, crystal structure manipulation, and surface affinity modification were proposed as the main methods for enhancing the osteogenic properties of hydroxyapatite^66–68^. The present study effectively obtains negatively charged G-IHA from golden pomfret bone, which possesses an intrinsic crystal defect that includes calcium deficiency and carbonate substitution. Without surface modification, G-IHA increases the osteogenic activity using its crystal structure. Here, we provide a new method for the synthesis of imperfect hydroxyapatite which can be potentially used in bone regeneration.

## Conclusion

**Fig. 8:**
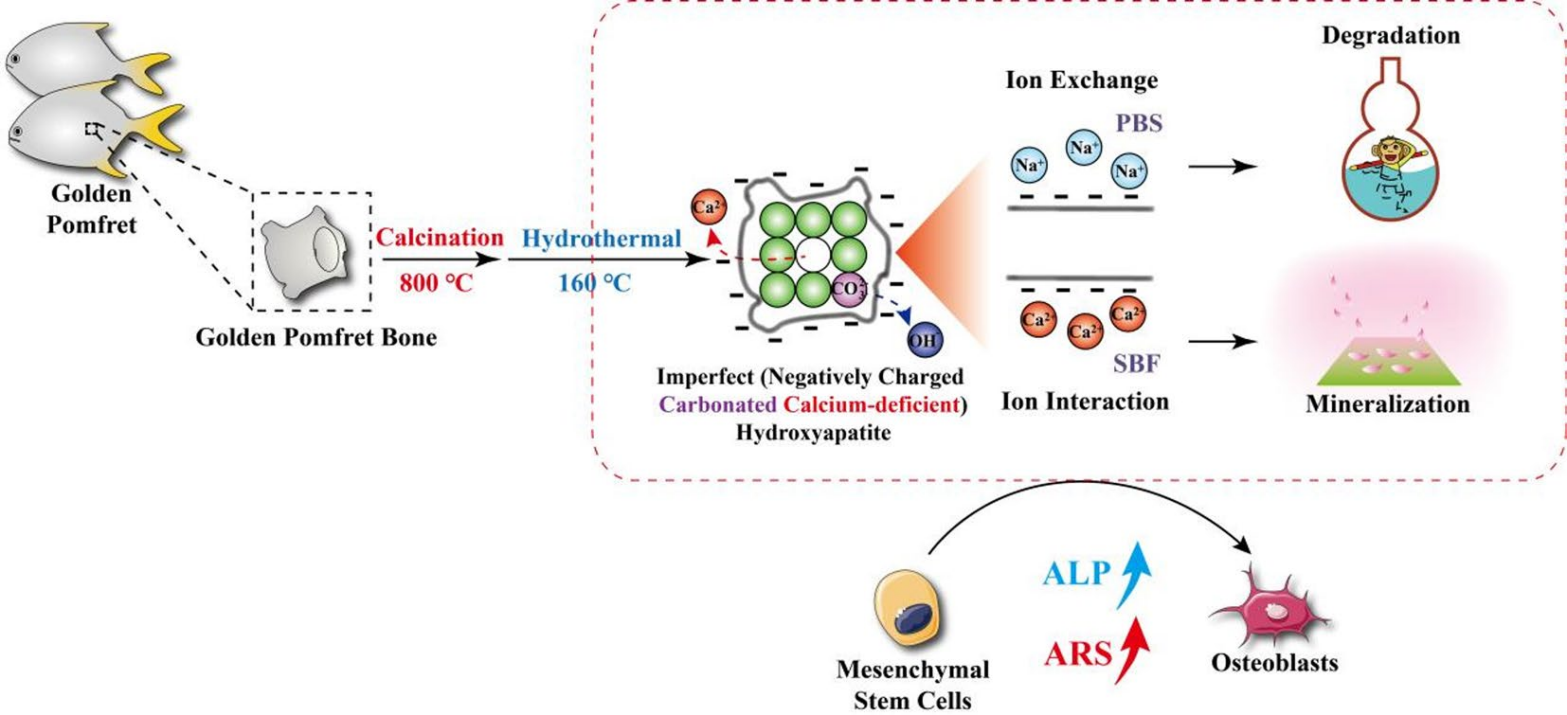
Illustration of the preparation method and action mode for golden pomfret derived hydroxyapatite.

In this study, the degradation, mineralization, and osteogenic abilities of golden pomfret bone-derived imperfect hydroxyapatite bioceramics (G-IHA) obtained from golden pomfret using a two-step calcination-hydrothermal method were investigated. In particular, carbonate substitution and Ca vacancies resulted in a negatively charged surface on the G-IHA. The negatively charged surface of the HA structure, facilitating the cation exchange and interaction, endowed it with superior degradation, mineralization, and osteogenic properties (Fig. 8). Consequently, G-IHA are likely to become novel and widely used bone graft materials, benefiting > 450 million patients with bone fractures worldwide.

Future research should focus on the production of injectable materials that can sustainably release G-IHA. Thus, G-IHA can implant to the fractures non-invasively and induce osteogenesis on their surface. In vivo investigations will be conducted in the future using different damage models of bone fractures to further verify the biological safety and clinical efficacy of G-IHA.

## Data Availability

The data that support the findings of this study are available from the corresponding author upon reasonable request.
